# Chimpanzees communicate to coordinate a cultural practice

**DOI:** 10.1098/rspb.2022.1754

**Published:** 2023-01-25

**Authors:** Zoë Goldsborough, Anne Marijke Schel, Edwin J. C. van Leeuwen

**Affiliations:** ^1^ Department for the Ecology of Animal Societies, Max Planck Institute of Animal Behavior, Bücklestraße 5a, Konstanz, 78467, Germany; ^2^ Animal Behaviour and Cognition, Department of Biology, Utrecht University, Padualaan 8, Utrecht, CA 3584, The Netherlands; ^3^ Department of Biology, University of Konstanz, Universitätsstraße 10, Konstanz 78464, Germany; ^4^ Behavioral Ecology and Ecophysiology Group, Department of Biology, University of Antwerp, Universiteitsplein 1, 2610 Wilrijk, Belgium; ^5^ Centre for Research and Conservation, Royal Zoological Society of Antwerp, K. Astridplein 26, B 2018 Antwerp, Belgium

**Keywords:** grooming handclasp, *Pan troglodytes*, communication, culture, coordination

## Abstract

Human culture thrives by virtue of communication, yet whether communication plays an influential role in the cultural lives of other animals remains understudied. Here, we investigated whether chimpanzees use communication to engage in a cultural practice by analysing grooming handclasp (GHC) interactions—a socio-cultural behaviour requiring interindividual coordination for successful execution. Previous accounts attributed GHC initiations to behavioural shaping, whereby the initiator physically moulds the partner's arm into the desired GHC posture. Using frame-by-frame analysis and matched-control methodology, we find that chimpanzees do not only shape their partner's posture (22%), but also use gestural communication to initiate GHC (44%), which requires an active and synchronized response from the partner. Moreover, in a third (34%) of the GHC initiations, the requisite coordination was achieved by seemingly effortless synchrony. Lastly, using a longitudinal approach, we find that for GHC initiations, communication occurs more frequently than shaping in experienced dyads and less in mother–offspring dyads. These findings are consistent with ontogenetic ritualization, thereby reflecting first documentation of chimpanzees communicating to coordinate a cultural practice. We conclude that chimpanzees show interactional flexibility in the socio-cultural domain, opening the possibility that the interplay between communication and culture is rooted in our deep evolutionary history.

## 1. Introduction

Human culture is catalysed by many forms of communication, ranging from active teaching to subtle cue-responding during turn-taking [1,2]. By means of communication, information—including cultural knowledge—can spread efficiently, resulting in a bolstering of the within-group homogeneity and between-group heterogeneity typical of cultural variation [3,4]. While pivotal to human culture, it is currently unknown whether non-human animal culture (henceforth ‘animal culture’) is similarly guided by communication. Animal culture has been defined in many ways [5,6], yet a shared feature across all definitions is that the corresponding behaviour needs to be transmitted via social learning, which is the process by which individuals obtain information through observation or interaction with others or their products [7]. Moreover, many scholars adhere to the definitional criterion that such social learning processes ought to lead to group-specific behaviours [8]. Following this definition, there is currently little doubt that many animal species possess, and live by, cultural traditions [9–11].

However, an outstanding question related to animal culture concerns the specific transmission mechanisms of their traditions. How exactly does acquired behaviour spread through groups of animals such that it becomes culture? An impressive body of experimental work has shown that animals learn new behavioural variations both individually and by means of social learning [8,12]. Additionally, there is a large body of evidence from the wild indicating that animals establish cultural traditions, ranging from vocal dialects in birds and whales [10,13,14] to arbitrary conventions in meerkats and primates [15–17].

Yet, what we currently do not know is how animals transmit cultures that solely exist by means of the interactions between individuals (henceforth ‘cultural interactions’). For instance, in humans, there are many cultural behaviours that ‘take two to tango’, such as the tango itself, but also many dyadic encounters like greeting exchanges and conversing [1,18]. Do animals have similar cultural interactions? And if so, how are they instigated and maintained? Moreover, we do not yet know whether animals actively communicate to uphold these interactive cultures. To our knowledge, most of the documented examples of animal culture concern instances in which an observer learns from an otherwise passive other. In other words, individual A copies the behaviour of individual B without individual B actively transmitting its cultural knowledge. The more active forms of cultural transmission are embodied in human pedagogy and teaching [4], yet while there are several indications that animals may teach as well [19–21], the consensus lies, at least in chimpanzees, with a minimal account of teaching in which an active role of the purported teacher has yet to be identified [22,23].

**Figure 1.**
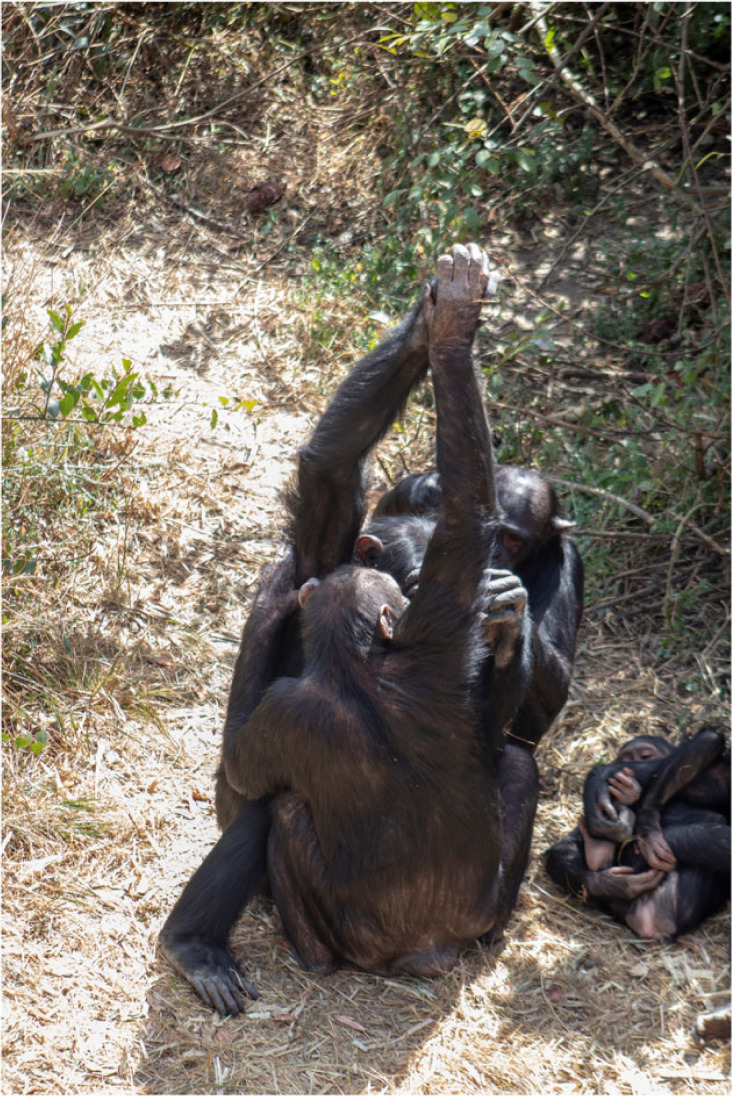
Moyo (back) and Tess (front) engaged in a palm-to-palm GHC (back-view: photo by Zoë Goldsborough).

Here, we investigate an enigmatic cultural interaction in chimpanzees—the grooming handclasp (GHC) [24]—to test whether chimpanzees may actively communicate to coordinate these interactions. In a GHC, two partners extend one of their arms overhead and clasp each other's extended hand at the palm, wrist, or forearm, while grooming each other with the other arm ([24–26]; figure 1). While the cultural nature of GHC has been firmly established by reports on inter-group differences in the form and frequencies of practices [26–30], little is known about the ways in which chimpanzees coordinate the execution of this cultural interaction, other than one individual (i.e. the initiator) physically shaping the body of the envisioned partner into the GHC posture [31]. To learn more about this coordination process, we studied the behaviours associated with the onset of GHCs by means of frame-by-frame analysis in a group of semi-captive chimpanzees in Zambia and compared the observed behaviours with matched-control (MC) windows (i.e. social grooming events of the same partners *without* GHC (*sensu* [32]). Consistent with reports evidencing intentional communication in social contexts like joint travel and play [33,34], we hypothesized that chimpanzees would not merely shape their partner into the typical handclasp posture [31], but actively communicate to coordinate their cultural practice. Finding support for this hypothesis would provide the first evidence for non-human animals to organize their cultural lives by means of proactive communication, akin to the human species.

## 2. Methods

### (a) Subjects

Subjects were 52 semi-wild chimpanzees (electronic supplementary material, table S1) at the Chimfunshi Wildlife Orphanage, Zambia, a sanctuary where chimpanzees live in Miombo woodland enclosures (size = 65 ha) where they can nest and forage independently but do receive daily feedings [26]. GHC has been frequently observed in the group for over 12 years [35]. The study was purely observational and approved by the Chimfunshi Research Advisory Board (Project: CWOT_ 2019C039), which evaluates studies both for feasibility and ethical procedures. Furthermore, the study strictly adhered to the guidelines as stipulated by Chimfunshi—a sanctuary accredited by PASA and adhering to the rules and regulations with respect to animal care and management as stipulated by the Zambia Wildlife Authority.

### (b) Collection and coding

Data were collected by ZG from 21 March 2019 to 1 August 2019 between 08.00 and 16.00 with handheld digital video cameras (Panasonic HDC-HS100). To capture GHC initiations, filming commenced as soon as two individuals approached one another. Filming continued if the individuals started social grooming (uni- or bi-directional) and lasted until they (i) had stopped grooming for over 30 s, (ii) started grooming another individual, or (iii) physically separated. A grooming bout was defined as running from the start of grooming until the moment one of the aforementioned ending conditions was met. A bout was considered a GHC bout if it contained one or multiple GHCs, and a regular grooming bout if no GHCs occurred.

**Figure 2.**
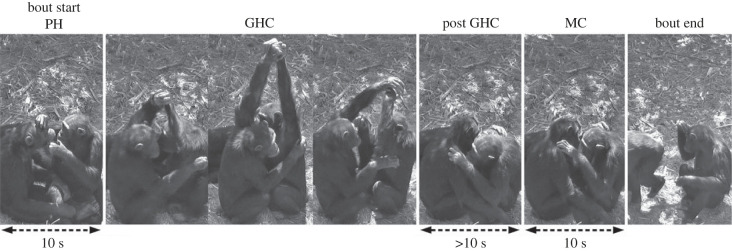
Side-view of GHC bout including the identified PH and MC period. The PHs and MCs were chosen to exactly match in terms of individuals, bodily positioning and activities (grooming) in order to identify the mechanisms by which GHC is initiated.

GHC bouts had either a *side* (optimal) or *back* (sub-optimal) view. If a 10 s pre-handclasp (PH) social grooming window was available before the first GHC in the bout (figure 2), we used the video for analysis. We only analysed the initiation of the first GHC in GHC bouts, because previous GHCs could possibly function as signals for subsequent GHCs. The start of a GHC was defined as the instance of handclasp above face level; the end was defined as the instance that physical contact of the arms was broken. A MC period (figure 2) was analysed to enable comparison of individual initiation behaviours across conditions [32]. The MC period was defined as a 10 s-window minimally 10 s after the last GHC occurrence in the bout, in which the same individuals had to be positioned in the same relative positions as during the GHC, while still engaging in social grooming. Additionally, initiations of regular, non-GHC bouts were opportunistically recorded (*n*_side+back_ = 23) to identify behaviours used in the initiation of regular social grooming bouts.

Videos were scored in ELAN [36] and behaviours were coded based on preliminary screening of the videos and established chimpanzee ethograms [37] (table 1; electronic supplementary material, videos). The majority of behaviours correspond to ethograms in other gestural studies (e.g. ‘elbow touch’, ‘hand touch’ and ‘head touch’ all correspond to ‘touch other’ by [38]). However, for our aim, we required more fine-tuned descriptions of gestures in the specific GHC initiation context, so, for example, we specify the location of the brief touch. A subset of 20% of the data was coded by two further observers to establish inter-rater reliability (IRR). Mean dyadic agreement was 0.833 for coding behaviours (range 0.81–0.89), and 0.973 for identifying the initiating individual (range 0.89–1; see electronic supplementary material for details).

**Table 1.** Ethogram of behaviour, with reference to electronic supplementary material, videos of behaviours.

behaviour	video	definition
elbow hold	S1	places own hand on elbow or arm of other, maintaining physical contact as arm of other moves
elbow touch	S2	briefly touches elbow or arm of other with own hand
hand grab	S3	grabs hand of other with own hand, maintaining contact as arm or hand of other moves
hand touch	S4	briefly touches hand of other with own hand
head move	S5	tilts head up or downwards
head touch	S6	touches (side of) head of other with hand, brief or prolonged contact
hold	S7	holds arm up in the air at peak of arm-raise movement, i.e. the raising of the upper arm with some flexion in the elbow
nosewipe	S8	swipes hand across or underneath nose in quick motion
raise	—	raises upper arm with some flexion of the elbow
self-scratch	S9	drags hand across body in long rough strokes
torso	S10	turns torso towards or away from other

### (c) Analyses

To determine the mechanisms underlying GHC initiation, we investigated the occurrences of 10 selected chimpanzee behaviours (table 1; electronic supplementary material, table S2) in a comparison between PH periods and their MC windows in video-recorded GHC bouts with the optimal vantage point (*side*-view, *n*_ph-mc_ = 94; *n*_ind_ = 33, *n*_dyads_ = 48, figure 2). In this sample, the mean number of bouts that an individual was involved in was 5.7 (range 1–28), with eight individuals only involved in one bout. All analyses were done in R v. 4.0.3 [39]. When necessary, non-parametric statistics were applied, including Bonferroni–Holm corrections for multiple testing [40].

In the PH–MC comparison, we only analysed those behaviours that occurred greater than or equal to five times in the PH and MC of GHC bouts (see electronic supplementary material, table S2). Furthermore, two behaviours (nosewipe and self-scratch) were excluded from this analysis, as they are known self-directed behaviours linked to increased arousal [41,42] and, as such, may also be produced in other contexts besides initiating a GHC interaction (though they may be inadvertent signals, revisited below). The behaviour ‘torso’ was also excluded as it is potentially an artefact of chimpanzees turning towards their partner as a necessary prelude for a grooming interaction. Additionally, given that social grooming occurred in both PH and MC windows by definition (figure 2 and ‘Collection and coding’), we did not consider the grooming behaviours themselves (see electronic supplementary material, table S2) as possible signals for GHC initiation. ‘Raise’, raising of the arm, was also not included, since this behaviour is mechanically necessary to perform a GHC and therefore occurs in every PH period. Based on our findings (see Results) and previous literature (e.g. [31]), we classified the behaviours identified in the PH–MC comparison into three types of GHC initiations: (i) *shaping*, (ii) *communication* and (iii) *synchrony*. We also conducted auxiliary analyses including *back* -view observations of GHC bouts (*n*_total_ = 133, *n*_individuals_ = 34, *n*_dyads_ = 57). In this larger sample, the mean number of bouts per individual was 7.7 (range 1–35), with seven individuals only involved in one bout.

To assess the flexibility of initiation sequences and presence of elaboration in GHC initiations, we used all *side*-view GHC bouts regardless of the presence of MCs (*n* = 114, *n*_individuals_ = 34, *n*_dyads_ = 58). In this sample, the mean number of bouts an individual was involved in was 6.5 (range 1–24), with eight individuals only involved in one bout. We considered the flexibility of GHC initiations by exploring variation in the start behaviour of the initiator as well as variation in their behavioural sequences in the PH period. An individual was considered the initiator of a GHC bout when they were either (i) the first one to produce a GHC-specific initiation behaviour or (ii) in the absence of these behaviours, the first to raise their arm for the GHC. Note that seven bouts have been dropped from this sample, as these were initiations where the initiator did not show any behaviours before raising their arm. We define elaboration as the use of new or additional behaviour after an initial behaviour did not lead to a GHC [43].

Lastly, we examined what factors could account for how a dyad initiated a GHC. Mothers play a more active role in the acquisition of the GHC by their offspring than other group members [26,28], largely through physically shaping their offspring in the GHC posture. Thus, we expect that GHCs between mother–offspring dyads are more likely to be initiated via physical shaping than GHCs between dyads that are not mother–offspring. Additionally, dyads with more experience engaging in GHC with each other could be better at coordinating the GHC interaction as a result of many repeated interactions. As such, experienced dyads might be less likely to initiate GHCs via physical shaping than inexperienced dyads. To test this, we employed a Bayesian categorical (multinomial) model on all *side*-view PH–MC bouts (*n* = 94). The outcome variable was the type of GHC initiation observed in a bout (*shaping*, *communication* or *synchrony*, with *shaping* as reference category), and the predictor variables were (i) days of experience (days since this dyad's first GHC, based on a longitudinal dataset of opportunistic GHC observations between 2007 and 2019, standardized such that 0 is no experience and 1 the highest number of days in the sample) and (ii) whether the dyad was a mother–offspring dyad or not. To account for dyadic or initiator preferences for specific initiation types, we included dyad and initiator ID as random effects. This model was fitted with the brms package v. 2.16.1 [44]. We set mild regularizing priors—normal(0,1) for estimates and exponential(1) for standard deviations—and performed a prior predictive simulation to visualize the priors. For running the final model, we used four chains, 10 000 iterations, and a credibility interval of 0.95. Our model was stable with large effective sample sizes (Bulk_ESS and Tail_ESS over 1000) and Rhat values equal to 1.

## 3. Results

We observed a total of 548 GHC initiations (of which 133 were recorded on video) during the five-month study period (electronic supplementary material, table S1). Of the 52 chimpanzees in the group, 39 individuals (including all adults) were observed to engage in GHC at least once.

### (a) Grooming handclasp-specific behaviours

Seven behaviours were observed more frequently in the PH compared to the MC context (Wilcoxon signed-rank: all *p* < 0.04, Holm-corrected; see electronic supplementary material, table S3). These behaviours were thus considered to be potential mechanisms leading to GHC interactions. Due to their physical nature, two of these behaviours (elbow hold and hand grab) corresponded to the documented practice of *shaping* ([31]; e.g. electronic supplementary material, video S11), while the remaining behaviours (elbow touch, hand touch, head move, head touch and hold) lacked any prolonged physical contact with the partner and were thus considered to be potential *communicative gestures* ([45]; e.g. electronic supplementary material, video S12). Auxiliary analyses including the sub-optimal *back*-view bouts supported our main analyses (all seven initiation behaviours significantly more present in PH than MC, all *p* < 0.02, Holm-corrected; see electronic supplementary material, tables S2 and S3).

**Figure 3.**
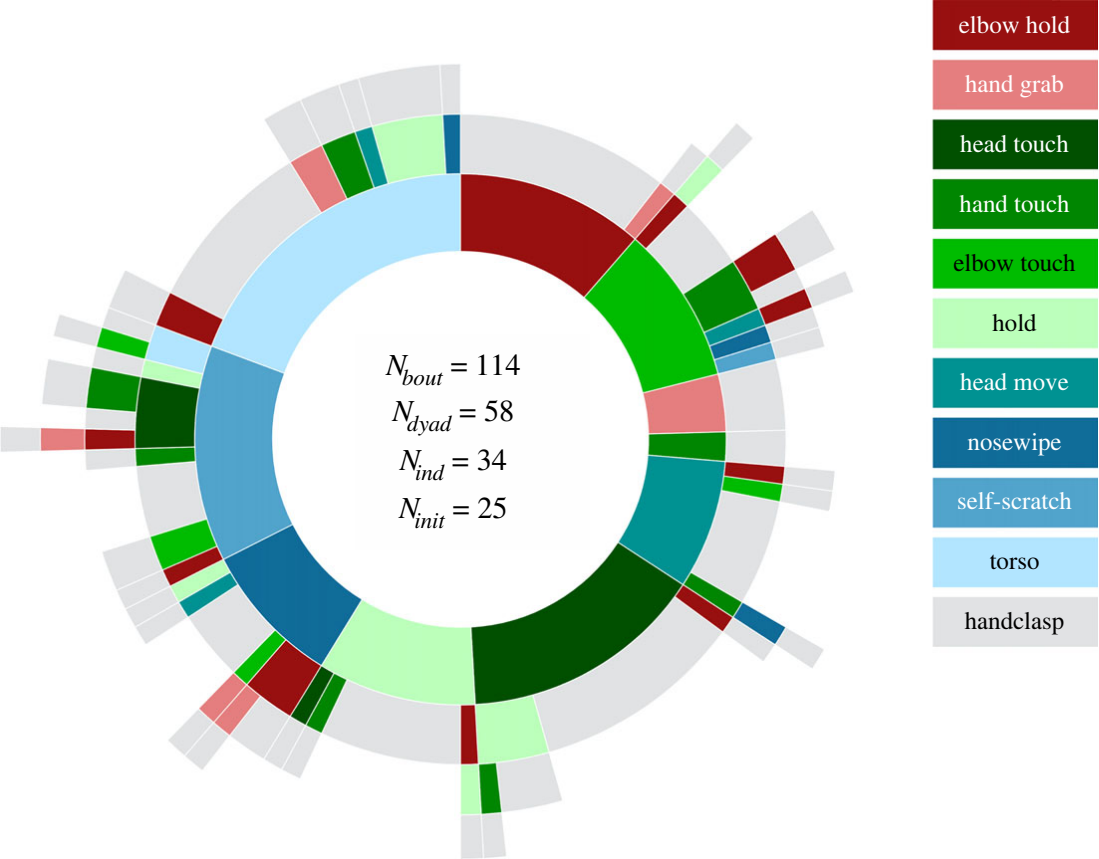
Sunburst [47] showing behavioural sequences (*n* = 114) by the initiator (*n* = 25) leading to GHC. Starting behaviours are depicted in the inner coloured circle, with the grey outer circle being the endpoint of the sequence (i.e. GHC). In order to consider the full flexibility of all types of GHC initiations, we also included the three *synchrony* behaviours (nosewipe, self-scratch and torso). An interactive version is available as electronic supplementary material, figure S13.

Gestures are here defined as bodily actions directed at a conspecific that are mechanically ineffective and result in a voluntary response from the recipient [45,46]. The five potentially communicative GHC initiation behaviours complied with this definition in being mechanically ineffective bodily actions resulting in voluntary GHC responses. Only for ‘head move’ we could envision partly covariation with other behaviours such as changing grooming posture or redirecting attention. ‘Elbow touch’, ‘hand touch’ and ‘head touch’ involved targeted physical contact from actor to recipient and were thus directed at a conspecific, and during ‘hold’ and ‘head move’ signallers faced their recipient in 100% of observed instances (*n* = 31 and *n* = 18, respectively). Fourteen of 15 individuals performing more than one GHC initiation showed variation in the start behaviour (i.e. the seven GHC-specific behaviours determined above) of their initiation sequences (binomial test: *p* < 0.001; also figure 3 and associated R-code).

Moreover, the gestures were produced in a goal-directed way, as indicated by the occurrence of *elaboration* in 29% of the cases where an initial gesture failed to initiate a GHC (*n* = 20 out of the 69 instances where gestures were used in the initiation, figure 3 and details below). Elaboration occurred after an average response waiting time of approximately 0.5 s and took the form of another gesture (*n* = 11), a shaping behaviour (*n* = 4), or a combination of both another gesture and a shaping behaviour (*n* = 5) before the GHC finally commenced. Taken together, these observations show that chimpanzees are capable and determined to (re-)transmit their motivation to engage in GHC when needed.

### (b) Grooming handclasp initiation types

In general, of the 94 PH/MC comparison bouts, 21 (22%) contained either one or both shaping behaviours (elbow hold, hand grab), 41 (44%) contained one or more of the five communicative gestures (elbow touch, hand touch, head move, head touch, hold) and no shaping behaviours, and 32 (34%) contained neither shaping behaviours nor potentially communicative behaviours. We labelled the third type of GHC initiation as *synchrony*, as the individuals appeared to commit to the GHC near-simultaneously. If any behaviour was scored during the PH window in the synchronous GHCs, these were either the previously mentioned self-directed behaviours, namely ‘self-scratch’ and ‘nosewipe’, or ‘torso’. These behaviours might signal to a partner that an individual is in a high state of arousal [42] or on the verge of initiating a grooming interaction [48], which the partner could respond to by raising their arm for a GHC. The reason that we do not consider these behaviours as GHC-specific signals, however, is that these three behaviours were relatively frequent in the initiation of regular grooming bouts as well, whereas two out of five communicative signals were entirely absent in the regular grooming bouts (only ‘hold’ and ‘head move’ both occurred once, and ‘head touch’ four times, see electronic supplementary material, table S2).

**Figure 4.**
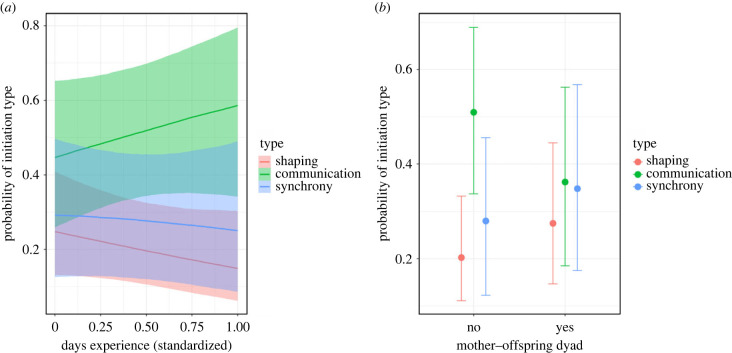
Probability of GHC initiation type in relation to (*a*) dyadic days of experience with GHC and (*b*) whether the dyad is a mother–offspring dyad or not. The lines in (*a*) and dots in (*b*) indicate the model fit of the multinomial model in table 2, with the shaded areas corresponding to the 95% credibility interval.

We found that the probability of a dyad initiating a GHC bout via communication rather than shaping increases when the dyad has more GHC experience (table 2 and figure 4*a*). Here, 91% of the posterior distribution lies above zero, which indicates that a positive relationship is expected to be observed 91% of the time. Additionally, mother–offspring dyads appear less likely to initiate a bout via communication (compared to shaping) than non-mother–offspring dyads (table 2 and figure 4*b*), with a probability of observing a negative relationship 89% of the time.

**Table 2.** Posterior mean estimates of multinomial model testing for the effect of GHC experience and mother–offspring kinship on type of GHC initiation. The parameter estimates are on the logit scale and are in relation to the reference category of the model, which is *shaping*.

parameter	estimate	CI_95_low	CI_95_high
communication–intercept	0.58	–0.28	1.45
synchrony–intercept	0.14	–0.91	1.09
communication–days experience	0.78	–0.36	1.90
communication–mother–offspring (yes)	−0.65	−1.72	0.38
synchrony–days experience	0.35	−0.84	1.52
synchrony–mother–offspring (yes)	−0.06	−1.20	1.14

## 4. Discussion

In this study, we set out to test whether communication may play a role in facilitating cultural practices in animals other than humans. Human culture is vastly nurtured by means of communication (e.g. verbal instructions on how to behave in the classroom), but whether such influences exist in the animal kingdom remains to a large part unknown. Here, we show that chimpanzees communicate to engage in one of their most enigmatic socio-cultural practices, the GHC [24]. In addition to physically shaping a partner into the GHC position—the hitherto only explanation for the coordination of the GHC—we identify goal-directed gestural communication as an additional mechanism by which chimpanzees initiate and coordinate their culturally bestowed handclasps.

While it is known that chimpanzees intentionally communicate to entice group members into desired responses [33,34,49], to date, such communication has not been reported in the context of cultural practices. The GHC is arguably one of the most convincing examples of animal culture for at least three reasons: (i) there is substantial variation with respect to its prevalence in groups of both chimpanzees and bonobos [24,50], (ii) within the chimpanzee groups that engage in the GHC, there is substantial and stable variation in the specific technique preferred [26–28,35] and (iii) the GHC does not require environmental input, making it a behavioural phenomenon that is independent on the local availability of materials unlike most other cultural traditions in chimpanzees (e.g. see [50,51]). The latter reason makes it less probable that the GHC is in fact a behaviour that is instigated and formed by non-cultural determinants [35], much like vocal cultures in birds [10] and cetaceans [52]. Unlike vocal cultures in birds and cetaceans, however, in the chimpanzees' handclasp case, the (non-vocal) communication is used to enact their cultural practice. Whether the chimpanzees’ communicative repertoires to coordinate their socio-cultural GHC behaviour are themselves shaped by cultural processes remains an interesting, yet outstanding empirical question [53].

Previously, it was known that chimpanzees solve the coordination problem inherent to handclasping by means of physically shaping the desired partner into the typical A-frame posture of the GHC [31]. Here it is important to note that this mechanism requires one adamant individual and at most a passive, yet non-declining partner. The ensuing process is consistent with described patterns of transmission: for a long period of time, typically there is one eager individual in a group who initiates most, if not all GHC interactions (e.g. chimpanzee ‘Georgia’, see [31,54]). However, this propagation mechanism (i.e. the proactively shaping of a willing partner) only covers the early stages of transmission—what mechanisms might sustain this socio-cultural tradition once there are more proficient group members? While shaping may still be involved, with two skilled and motivated partners the GHC becomes more fluent and bidirectional. In other words, our finding that chimpanzees also use non-physical means to initiate a handclasp identifies an active partner response (otherwise the GHC would not ensue) and implicates a willingness from both partners to engage in this cultural practice.

In this light, it is worth highlighting that the active involvement of the partner complements the initiated GHC sequence by virtue of which the interaction may be considered as a joint action—at least in comparison to shaping interactions. With two voluntarily acting partners (i.e. one chimpanzee showing this by initiating, the other chimpanzee showing this by responding) in a tightly coordinated interaction (i.e. there is only a small window in space–time where the arms can clasp), the GHC may offer a fruitful context in which to study joint commitment and perhaps even joint/shared intentionality. Future research in this domain may benefit by extending the scope of GHC scrutiny from initiations (this study) to interactions during and revolving around the ending of the interaction [55].

Our GHC analyses revealed a third coordination process which we coined *synchrony* due to its indiscernible execution. In these cases, the GHC coordination was fluent to the extent that no shaping or communication was required to accomplish it. We conjecture that perhaps repeated GHC engagement attunes partners' behaviour to the extent that a subtle indication, be it behaviourally (e.g. a nose wipe, first indications of an arm's raise) or embodied by contextual factors (e.g. the order or duration of the ongoing grooming session), suffices to achieve coordination. Yet, our analyses did not indicate that more experienced dyads showed more ‘synchronous’ GHC interactions. It may be that our sample was too dispersed to obtain a reliable indicator of ‘experience’ (i.e. spanning relatively short observation windows between 2007 and 2019). Alternatively, it might be that our ethogram lacked the resolution to identify coordination behaviours that preclude the ‘synchronous’ label. Finally, it may be that other factors than mere experience contribute to partners becoming fluent at achieving fine-tuned coordination, for instance individuals' motivation and capacity to pick up on social cues.

The findings that (i) communication is used more frequently as GHC initiation strategy than shaping in experienced dyads, and (ii) shaping is more frequent in mother–offspring dyads than in non-mother–offspring dyads, are largely consistent with ontogenetic ritualization playing a role in the development of this communicative strategy [56]. This theory suggests that some behaviours can start to function as communicative signals through mutual anticipation of both partners following repeated shaping interactions. Inexperienced GHC dyads, such as a mother with younger offspring, may use physical shaping behaviours like ‘elbow hold’ and ‘hand grab’ to initiate a GHC. Over time, the offspring learns to anticipate the hold or grab and starts raising their arm at a touch of the elbow or hand, without requiring it to be held. However, not all gestures linked to the GHC initiation can be explained through ontogenetic ritualization: for instance, ‘hold’ and ‘head touch’ are unlikely to have arisen in this way because they include limited to no physical contact, and many other gestures in the chimpanzee repertoire cannot be explained as such [49]. Moreover, chimpanzees do not only handclasp with their mothers [26] and thus seem to need a form of generalization regarding the initiation communication in order to successfully coordinate a GHC with others. Especially with respect to the particular forms of handclasping that the chimpanzees converge on (e.g. palm-to-wrist), be it within family units [30] or more widely at the group level [29], the question arises how exactly the GHC configurations are shaped and shared within groups.

If ontogenetic ritualization is the driving force of the development of social customs in chimpanzees, one would expect to find idiosyncratic gestures: different dyads employing different gestures for the same purpose. However, in the case of GHC, this would not be very probable since there are only limited ways to shape another individual's body into the necessary A-frame posture typical of the GHC. Therefore, even following the theory of ontogenetic ritualization, within the GHC context, different dyads could end up using similar gestures to initiate a GHC, like ‘elbow touch’ and ‘hand touch’. Of the remaining gestures, ‘hold’ has previously been named as a behaviour to invite another individual to handclasp in chimpanzees [57] and bonobos [58]. The gesture ‘head touch’ could be a soliciting act [23] to draw a partner's attention and indicate an intent to groom or start an interaction. In this specific group of chimpanzees, GHC is so common that we rarely observed regular grooming bouts without GHCs. Although we could not observe the chimpanzees at all times, this might indicate that GHC grooming is the most frequently used form of grooming in this group. In this context, ‘head touch’ might have come to serve, fortuitously, as a signal for GHC initiation in this group.

Finally, our results provide new insights into how chimpanzees may coordinate their actions in general. The GHC is a social activity that requires coordination for successful execution. Chimpanzees cooperate [59], but not much is known about the ways in which they coordinate their joint efforts [60]. In experimental settings, some chimpanzees used location-enhancing behaviours (e.g. bodily positioning, touching and peering) [61], or generic gestures (e.g. arm fling, clapping, banging on panels; [62]) to recruit their conspecific partners for (re-engaging in) a cooperative task. While these behaviours can be interpreted as communicative acts to overcome coordination problems (for alternative interpretations, see [60,63,64]), to date, it remains an interesting question why chimpanzees seem to use communication so little in contexts where it seems so obvious for humans [65].

Our findings show that chimpanzees communicate to coordinate a naturally occurring cultural practice. The socio-cultural interaction was not shaped by just one invested individual [31,54], but when the initiator communicated its desire to engage in the GHC (e.g. by holding out its flexed arm at face level in front of the desired partner), active compliance in the form of a complementary action by the partner was required to accomplish the interaction. Thus, when communicated, a GHC initiation appeared to function as an invitation to join in a cultural practice. Following up on reports showing that chimpanzees and bonobos use communication during interactions like joint travel and social play [33,66,67], we conclude that chimpanzees communicate to coordinate a cultural practice, and propose that our findings warrant future scrutiny of the GHC as a putative case of joint intentionality in great apes [55,66,68].

## Data Availability

All code and data used for analyses are available [69]. The data are provided in the electronic supplementary material [70].
